# Editorial: Roles and mechanisms of parasitism in aquatic microbial communities

**DOI:** 10.3389/fmicb.2015.00446

**Published:** 2015-05-12

**Authors:** Télesphore Sime-Ngando, Kevin D. Lafferty, David G. Biron

**Affiliations:** ^1^Université Clermont Auvergne, Université Blaise PascalClermont-Ferrand, France; ^2^Laboratoire Microorganismes: Génome et Environnement, Centre National de la Recherche ScientifiqueAubière, France; ^3^Western Ecological Research Center – The United States Geological Survey, Marine Science Institute, University of California, Santa BarbaraSanta Barbara, CA, USA

**Keywords:** parasitism, hyperparasitism, ecology, food web dynamics, microbial communities

Our research topic on the roles and mechanisms of parasitism in aquatic microbial communities should be of broad interest, given that there are probably more parasitic species than free-living ones (Windsor, 1998), a hypothesis increasingly supported by next generation sequencing technologies of microbial taxa (Sime-Ngando and Niquil, 2011). We know little about the parasites of microbes, but recent research suggests that they affect food-web dynamics, biogeochemical cycling, the functioning of ecosystems and related services, and host evolution. Furthermore, several new research topics, such as interactomics, molecular dialogue, host manipulation by parasites (Cézilly et al., 2014; Biron et al., 2015), “beneficial” parasites (Roossinck, 2011; Parfrey et al., 2014), priming of the host immune system (Llewellyn et al., 2014), microbiomics (Llewellyn et al., 2014), and tripartite symbiosis (Rohwer and Thurber, 2009; Gleason et al., 2014), are filled with interesting questions. We ordered the thirteen papers in this issue according to the biological complexity of parasites, from phages-bacteria, phage-bacteria-animal, microparasite-microbe, microparasite-microhyperparasite-microbe, microparasite-animal to macroparasite-microbe interactions.

New findings in the papers include the conceptualization of viral lifestyles and the extension of their role as microbe killers, cell partners, or metabolic manipulators (Sime-Ngando, 2014). These relationships have applications for economics and conservation. For instance, shore-based abalone aquaculture can discharge pathogens like the intracellular bacterium *Candidatus Xenohaliotis californiensis* (WS-RLO), with potential impacts to wild abalone (Lafferty and Ben-Horin, 2014). However, a novel bacteriophage now infects the WS-RLO, improving the survival of infected abalone and thereby offering a potential tool for population management via phage therapy (Friedman et al., 2014). The importance of such tripartite interactions relates to Gleason et al. (2014) argument that parasites of parasites may increase the complexity of food webs, and play significant roles in suppressing diseases of animals, plants, or algae. The ecological importance of such disease dynamics is illustrated well by new quantitative data and modeling that shows how during blooms of inedible algae in freshwater lakes, (i) chytrid parasites of phytoplankton are able to shape aquatic ecosystems by altering sinking fluxes or determining system stability (Kagami et al., 2014), and (ii) divert about 20% of primary production to edible zoospores that comprise 50–57% of the zooplankton diet (Rasconi et al., 2014). This work is remarkable given how challenging it is to diagnose parasites of microbes in natural systems (Karpov et al., 2014). Economic incentive for improved diagnosis stems from the effects of infection dynamics on commercial-scale algal monocultures for bioenergy and chemical production (Carney and Lane, 2014). Several topics in this collection deal with microbial parasites and the microbiome of fishes and animals, demonstrating, for example, that viral, prokaryotic and small-eukaryotic parasites affect conservation and food security (Gozlan et al., 2014). For instance, indigenous microbiota affects innate and adaptive immunity, fish digestion, and nutrient metabolism (Llewellyn et al., 2014). The extent to which aquatic microbes differ from other small eukaryote communities is highlighted by Parfrey et al. (2014) who use high-throughput sequencing to consider how microbes in the mammalian gut reflect both host phylogeny and diet, and are distinctive from those in aquatic and terrestrial habitats. The microbiome might even influence host behavior as a result of the molecular crosstalk between a manipulative parasite and its host, disturbing the synthesis of neuroactive molecules (Biron et al., 2015). We end with Cézilly et al. (2014), who consider the hypothesis of conflict vs. cooperation in host manipulation, and provide empirical evidence that microorganisms can have synergistic and antagonistic interactions with co-occurring parasites.

We hope the contributions to this collection bring a new focus to the aquatic sciences. Microbial interactions are clearly important and largely unknown. There are still methodological barriers to assessing prokaryotic and eukaryotic parasites of aquatic microbes (Sime-Ngando and Niquil, 2011), although recent advancements provide new opportunities (Marano et al., 2012), which we expect will lead to, a predictive understanding of the role of parasitism in aquatic systems in particular, and of aquatic ecosystem functioning in general.

## Conflict of interest statement

The authors declare that the research was conducted in the absence of any commercial or financial relationships that could be construed as a potential conflict of interest.
